# Supplementary material for: Are carbon emissions trading and green financial instruments synergistic? -Comprehensive quantitative research based on content analysis
Source: PLoS One. 2024 Mar 7;19(3):e0298601. doi: 10.1371/journal.pone.0298601 (PMC10919601; doi:10.1371/journal.pone.0298601)
Supplement: S1 Data — (ZIP) [file pone.0298601.s001.zip › Data/地级市Z_F工作报告.zip!/─■╧─/╬Γ╓╥╩╨/└┤╫╘╣1⁄2╓┌║┼í╛┬φ┐╦╩2╛▌═°í┐_2010.docx]

各位代表：

现在，我代表市人民政府向大会作政府工作报告，请予审议，并请市政协委员和其他列席人员提出意见。

一、2009年工作回顾

2009年是新世纪以来我市经济社会发展较为困难的一年，也是全市上下积极进取，奋力开拓，在危机中逆势求进的一年。一年来，在自治区党委、政府和市委的正确领导下，我们深入学习实践科学发展观，紧紧围绕“保增长、保民生、保稳定”的要求，和全市人民并肩携手，坚定信心，迎难而上，圆满完成了市三届人大二次会议确定的各项目标任务，在全面建设小康社会的道路上又迈出了坚实一步。

(一)全力以赴保增长，主要指标完成好于年初预期。预计完成地区生产总值180.6亿元，增长12.6%；全社会固定资产投资165亿元，增长25%；地方财政一般预算收入12亿元，增长24.1%；城镇居民人均可支配收入12642元，增长10%；农民人均纯收入4405元，增长8%；社会消费品零售总额44.5亿元，增长18.4%；单位GDP综合能耗下降8%；主要污染物二氧化硫削减3.8万吨，化学需氧量削减1.4万吨，分别完成目标任务136%和116%。

——工业经济逆势回升。针对国际金融危机对经济生产的严重冲击，及时果断地出台政策措施，从加强煤、电、运力协调，落实国家税收减免政策，搭建新的融资平台，完善园区基础配套，支持新上项目建设等方面，帮助企业应对危机，稳固生产，在较短时期内有效遏制了工业大幅下滑的态头，实现了工业整体回升，破浪前行的有利局面。预计完成工业总产值270亿元，规模以上工业增加值71亿元，增长13%；地方规模以上工业企业完成增加值40亿元，增长30%。开工建设投资千万元以上的项目72个，庆华集团15万吨甲醇、吴忠卷烟厂技改扩建等40个项目建成投产，宁夏发电集团风电二期、华盈矿业镁合金一期工程等项目快速推进。新设太阳山盐、同、红(区)工业园，园区主干道路建成通车。“两城五园”配套设施进一步完善，承载带动能力不断增强，新增入园企业46家。

——农村经济稳步发展。预计完成农业总产值60亿元，增长6%。当年种植优质粮食90万亩，粮食总产8.3亿公斤；奶牛存栏15.1万头；肉牛出栏30.2万头；羊饲养量501万只；新增设施农业5.4万亩、甘草23.2万亩、饲草11万亩、高酸苹果3.1万亩、红枣10万亩、酿酒葡萄5.4万亩。农业“十大基地”建设扎实推进。市级以上农业产业化重点龙头企业达到132家，农民专业合作经济组织发展到310个。全年输出劳务24万人次，实现收入14.7亿元。当年新增造林绿化面积51万亩，栽植各类树木6000万株，造林质量和成活率是最好的一年，被评为“全区林业生态建设先进市”。农业基础设施进一步改善。高标准、大规模推进农田水利基本建设，治理农田89万亩，建设畦田97万亩，改造中低产田14万亩，五个县(市、区)全部获自治区“黄河杯”奖项，实现历史性突破。其声势之大、成效之好、获奖之多是历史少有，居全区第一。

——第三产业更趋活跃。预计完成第三产业增加值55.8亿元，增长11.8%。城乡市场持续繁荣，消费稳步增长。实施“双百市场工程”、“万村千乡市场工程”、“三新工程”， 新建和改造提升农家店261个，新建扩建各类市场25个，涝河桥牛羊肉市场改扩建、东郊市场迁建进展顺利。销售各类“家电下乡”产品3万多台(件)，销售额6143万元，兑付补贴842.7万元。食品加工产业发展加快，完成产值88亿元，增长17%。制定出台了促进房地产业快速健康发展的十一条措施，组织开展“2009魅力吴忠住房博览会”、“滨河新区房地产推介会”等活动，完成房地产投资26.9亿元，增长141%。启动了青铜峡黄河大峡谷旅游区规划建设，青铜古镇等一批重点旅游项目开工建设。成功举办中国(吴忠)文化旅游节，吴忠市被评为“2009年度中国旅游竞争力显著提升城市”，全年接待游客140万人次，旅游收入突破5亿元，增长17.6%。

——“两大任务”成效显著。把强力实施招商引资和上争资金“两大任务”，作为积极应对危机冲击，快速突破投资瓶颈，拉动社会投资和经济增长的重大举措，充分调动各级部门和社会各方面的积极性，千方百计争项目、引资金。中国节能投资公司、中国国电集团、中国建材集团、浙江盾安等一批国内大企业落户吴忠。全年共实施招商引资项目234个，计划总投资483.6亿元，实际到位资金120亿元；落实中央及自治区各类项目1499个，争取资金37.2亿元。不仅有力地支撑了全市经济社会发展，在危机与困难中开拓了新局面，而且激活了理念，历练了干部，凝聚了力量，点燃了干事创业的激情。

(二)率先打造黄河金岸，城乡建设大步推进。按照“打造黄河金岸、建设水韵吴忠”的要求，以“三城联创”为载体，加快推进城乡建设。全年实施城乡建设项目346项，完成投资70亿元，增长46%，投资规模创历年之最。市区西移东扩步伐加快，建成区面积扩大到58平方公里。全长68.5公里的滨河大道率先在全区建成通车。教育园区一期、新月广场建成投入使用，“两馆一中心”、黄河公路大桥、黄河楼等沿黄城市带地标性建筑项目进展顺利。101省道同心大道南北延伸段、团结路等道路改扩建工程全面竣工。组织实施城市提质增绿工程，市区绿地总面积达到2.2万亩，城市绿地率、绿化覆盖率分别达到36%和38%，人均公共绿地达到17平方米。实施城市防洪排涝工程和罗家河拓宽改造等工程，建成湿地湖面15个，新增水面1.3万亩。吴忠黄河湿地公园被确定为国家湿地公园。盐池县老城改造与新区建设、同心县城新区建设、红寺堡城区扩展与生态建设等快速推进，滚红高速公路、211国道白土岗至太阳山高速公路(半幅)建成通车。新建塞上农民新居示范庄点9个，综合整治旧村庄72个，改造危房危窑6720户。新建改建农村道路1132公里，是2008年的3倍。新农村建设在自治区考核验收中名列第一。

(三)统筹社会事业发展，民计民生不断改善。自治区10项民生计划和年初承诺为民办理的13件实事全部完成。教育资源不断优化。实施了中小学校舍安全工程，新建、迁建、扩建中小学校61所，撤并整合农村中小学校63所，调整搬迁市区中小学7所，新设开元、盛元两所城市小学，缓解了“大班额”、入学难问题。全市补充特岗教师826名。盐池县在全区山区县中率先实现教育强县目标。公共卫生事业显著加强。续建了吴忠市中医院、改扩建青铜峡市医院、盐池县医院，扩建乡镇卫生院20所，利通区建成国家中医药特色社区卫生服务示范区。甲型H1N1流感疫情得到有效防控。计划生育“星级乡镇”创建活动扎实推进，人口自然增长率控制在9.82‰，出生政策符合率达到93.99%。文化体育事业稳步发展。编辑出版《新中国60年吴忠市文学艺术精选作品大系》，组建吴忠市文化艺术团，编排精品剧目《水韵吴忠》，利通区高闸镇(舞蹈)、金积镇(社火)、板桥乡(舞龙)被命名为“中国民间文化艺术之乡”，“老毛手抓”、“张家枪”被列为自治区第二批非物质文化遗产。成功承办第三届中国(吴忠)中小学生书法节，被命名为全国中小学生书法节首创地。举办了全市第二届运动会。全民创业力度加大，建成各类创业园29个，创办小企业630个，新增就业岗位7400多个，发放小额担保贷款1.3亿元，新增城镇就业1.2万人，城镇登记失业率稳定在4.3%以内。社会保障覆盖面进一步扩大。城镇六大保险参保人数达到43.8万人，城乡低保、困难学生救助、孤残救助、“五保”集中供养得到较好落实，高龄低收入群体全部享受老年基本生活津贴，新型农村合作医疗参合人数87.5万人，参合率达94%。在盐池县启动农民养老保险试点。积极争取自治区补助资金，解决38家国有关闭破产和困难企业退休职工医疗保险问题。建成经济适用住房21.4万平方米、廉租房750套。在全区率先完成征地补偿费清理工作，兑付资金1.84亿元。搬迁安置移民1万户4.3万人。完成10处农村安全饮水工程，解决13万人饮水安全问题。全面贯彻落实民族宗教政策，民族团结、宗教和顺。切实维护社会稳定，深入开展大接访和矛盾纠纷大排查、大调处活动，信访积案化解工作取得明显成效。不断深化基层平安创建活动，公众安全满意度居全区五市之首。同心县摘掉了“全国毒品重点整治地区”帽子。安全生产、应急管理和防震减灾不断加强，人防、档案、外事、统计、物价、广播电视、妇女儿童、残疾人事业、住房公积金等工作有了新的发展。国防动员、军民共建和双拥工作取得新成绩。

(四)深入推进各项改革，体制机制呈现新的活力。在全区率先开展省直管县改革试点工作，积极稳妥地推进红寺堡区设立和调整理顺利通区行政管理职能改革，市直部门机构优化合并全面完成。全面启动乡镇机构改革。推行义务教育阶段阶段教师绩效工资改革，开展名师名校名校长评选表彰活动，激发教育发展活力。积极推行政府投资项目代建制，设立金融办、城乡建设投资公司和开源信用担保中心，努力缓解融资难、贷款难问题。深化财政制度改革，推广使用公务卡结算制度改革试点，集中清理和整顿行政事业性收费项目，深入开展“小金库”专项治理。率先在全区启动建立保障和促进科学发展土地管理新机制试点工作，积极探索农村土地经营权流转的新思路，取得了土地适度规模经营的积极成效。严格耕地保护制度，确保了占补平衡。

(五)切实加强政府效能建设，服务能力不断提升。在各级政府和部门扎实开展学习实践科学发展观活动，开展“四论四比四争”主题实践活动，取得积极成效。全面推进依法行政工作，推行政务公开、服务承诺、过错追究、首问负责和限时办结等制度，积极探索建立科学、合理的绩效评估机制。继续优化政府职能，精减审批事项，开展效能监察，推行高效便捷服务，全年共取消、转移、下放政府职能68项，强化78项，清理各类授权行政许可事项388项，取消60项。协调工商、税务、质监等区属部门进驻政务大厅，服务领域进一步拓宽。加大政府投资项目审计监督力度，资金使用效益不断提高。政务环境、司法环境、金融环境进一步优化，政府公信力和群众满意度不断提升。广泛听取各方意见建议，自觉接受社会各界监督，定期向人大、政协报告和通报重要工作情况，办理人大代表议案、意见和建议80件，政协委员提案302件。

各位代表！2009年非同寻常，我们共同经历了新中国成立六十周年的巨大喜悦，共同应对了新世纪以来最为严峻的发展挑战，共同开创了“保增长、保民生、保稳定”的大好局面。成绩来之不易，经验弥足珍贵。我们深深体会到：越是困难，越要坚持科学发展。我们把学习实践科学发展观活动作为破解危机的信心之源和力量所在，坚决贯彻落实中央、自治区和市委的决策部署，勇于担当，敢于突破，沉稳果断地狠抓关键，攻克难点，突破重点，努力保持了跨越发展的良好势头；越是困难，越要突出发展工业。我们把工业作为应对危机、保持增长的关键，攻坚克难，避害趋利，及时果断地采取一系列稳生产、保运行的有力举措，力促工业经济企稳回升，为整体经济增长发挥了重要作用；越是困难，越要注重借力发展。我们把“两大任务”作为争取外力、激活内力的关键举措，激发各方面的动力谋求更大突破，在非常之年发挥了非常之效；越是困难，越要不断夯实基础。我们着眼长远发展，以大投入、大气魄、大手笔加快黄河金岸建设和工业园区发展，优化基础环境，提升城市魅力，形成了新的发展优势；越是困难，越要大力改善民生。我们把民生作为最大的政治，倾斜财力采取实实在在的举措，实现了民生问题在困难时期得到更为有力的解决；越是困难，越要深化改革创新。我们把改革创新贯穿于应对危机、加快发展的全过程，在体制、机制上求突破、增活力，在重点领域和关键环节谋创新、促发展，推进改革与发展的良性互动；越是困难，越要全力维护稳定。我们把维护稳定作为第一责任，积极化解社会矛盾，加强社会管理，为发展创造了安定和谐的环境；越是困难，越要广泛凝聚民力。我们充分发挥社会主义的制度优越性，紧紧依靠人民群众，集中力量办大事，全市各个方面和各族人民面对困难不退缩、敢争先，打赢了一场齐心合力、共克时艰的特殊战争，取得了可喜成就。对此，我们要倍加珍惜，认真总结，不断发扬。

各位代表！回顾过去的一年，我们欣慰地看到，经过各族人民的共同努力，全市经济更加发展，社会更加进步，人民更加富裕，各项事业更加欣欣向荣。这是自治区党委、政府正确领导和市委统揽全局、科学决策的结果，是市人大、市政协依法监督、大力支持的结果，是全市各族干部群众锐意进取、艰苦奋斗的结果。在此，我代表市人民政府，向为吴忠发展付出辛勤劳动的全市各族人民，向给予政府工作大力支持的人大代表、政协委员、各民主党派、工商联、无党派、各人民团体、离退休老同志和社会各界人士，向为吴忠改革发展稳定做出积极贡献的公安干警、驻吴人民解放军、武警和消防官兵，向所有关心、支持吴忠发展的中央及区属驻吴单位，表示崇高的敬意和诚挚的感谢！

在肯定成绩的同时，我们也清醒地看到，全市经济社会发展中的一些固有矛盾还未根本消除，一些新的问题还在产生容不得半点松懈和停滞，需要以更大的魄力去推进解决。主要表现在：一是经济总量偏小，财政基础薄弱，发展水平仍然较低，产业结构不够合理，发展方式比较粗放。二是区域经济发展不够协调，城乡二元结构特征明显，城市化水平仍然较低，小城市、大农村的问题较为突出，山区贫困面还比较大，扶贫攻坚任务依然艰巨。三是工业受到的冲击仍未消除，支撑工业发展的大项目不多，企业竞争力不强。四是现代农业规模化发展速度不快，标准不高，特色农产品缺乏市场竞争力。五是一些人民群众关心的热点、难点问题还没有解决好，社会保障水平有待进一步提高。六是社会管理和公共服务仍不适应新形势、新任务的需要，一些部门效率不高、落实不力、服务不优的问题依然存在，与人民群众的要求还有差距。我们将在今后的工作中下更大气力，采取更加有力的举措，认真加以解决，不辜负人民的期待和重托。

二、2010年工作总体要求

2010年，是完成“十一五”规划的最后一年，也是必须实现“三年上台阶”的关键年。做好全年的各项工作，对保持经济平稳较快发展，夯实跨越式发展基础，顺利启动“十二五”规划，具有十分重要的意义。

纵观当前发展形势，新的一年仍然面临着不同的挑战与机遇，面临着新的困难与希望。虽然宏观经济企稳回升的基础仍不稳固，各种不确定因素依然很多，但经济发展的基本面和长期向好的趋势没有改变。我们完全有信心、有条件、有能力克服困难，战胜挑战。我们的信心和力量，来自于中央、自治区和市委对形势的科学判断和准确把握；来自于国家继续实施积极的财政政策和适度宽松的货币政策，国家启动实施西部大开发战略第二个十年规划，国务院《关于进一步加快宁夏经济社会发展的若干意见》及其《分工方案》等一系列利好政策蕴含的强大机遇；来自于工业化、城市化和社会主义新农村建设加速推进中形成的巨大市场需求；来自于我市丰富的矿产资源、土地资源、光能风能资源、农产品资源和劳动力资源等要素支撑；来自于市委三届四次全会以来提出并实施的一系列推进科学发展、跨越发展的战略举措和不断培育打造的一系列新的发展优势；来自于市委、人大、政府、政协等各个方面紧密团结，协调一致开展工作形成的坚强合力；来自于深化行政管理体制改革和优化政务、司法、金融环境等改革创新工作带来的持续活力；来自于全市干部群众盼发展、求跨越的强大精神动力和越干越想干、越干越会干的干事创业劲头。只要我们坚决贯彻落实中央、自治区和市委的决策部署，紧紧依靠全市各族人民，就没有克服不了的困难，就一定能够夺取应对危机、实现又好又快发展的最终胜利。

根据市委三届七次全体(扩大)会议精神，政府工作的总体要求是：全面贯彻党的十七大、十七届三中、四中全会精神和中央、自治区经济工作会议精神，以邓小平理论和“三个代表”重要思想为指导，深入贯彻落实科学发展观，继续把保持经济平稳较快发展作为首要任务，把转变发展方式和结构调整作为主攻方向，以项目为抓手，以投资为突破，以改革为动力，更加注重产业优化升级，更加注重统筹城乡发展，更加注重生态文明建设，更加注重社会建设和改善民生，更加注重社会和谐与维护稳定，更加注重政府效能提升，奋力开创全市跨越式发展新局面。

主要预期目标和约束性指标是：地区生产总值增长13%；地方财政一般预算收入增长15%；全社会固定资产投资增长25%；社会消费品零售总额增长15%；城镇居民人均可支配收入增长8%；农民人均纯收入增长7%；人口自然增长率控制在10.6‰以内，城镇登记失业率控制在4.3%以内，单位GDP能耗下降5%，化学需氧量和二氧化硫排放总量分别下降7%和8%。确立上述目标，综合考虑了发展的需要和可能。对于吴忠这样一个处于社会主义初级阶段较低水平的欠发达地区，保持持续较快的经济增速符合人民增收、改善民生和维护稳定的要求，符合科学发展、跨越式发展的需要。确立13%的地区生产总值预期目标是必需的，也是经过努力能够实现的。

为此，做好今年的政府工作，实现上述目标，必须把中央、自治区精神与我市实际有机结合，聚精会神搞建设，一心一意谋发展，保持经济持续快速发展的良好势头；必须把转变发展方式与提升产业竞争力有机结合，加快发展风电、光伏发电等新能源产业，大力发展低碳经济，促进优势特色产业加速扩张；必须把增加投资、扩大消费与促进出口有机结合，抓住重大项目，增强消费信心，借助自治区加快发展内陆开放经济和打造“三大口岸”的东风，把出口贸易做上去，努力促进“三驾马车”同时发力；必须把激活内力与借助外力有机结合，用改革破解发展难题，用开放拓展发展空间，把“两大任务”作为加快发展的“第一要事”，充分激发各各方面的能动作用；必须善于把经济建设和生态建设、社会建设有机结合，突出可持续发展，突出以人为本，办好社会事业，让全市各族人民更多地分享改革发展成果！

三、2010年主要任务

今年政府工作面对的形势十分严峻，肩负的任务异常艰巨，必须有打大仗、打硬仗的充分准备，统筹兼顾，勇于突破，重点抓好以下几个方面的工作：

(一)继续狠抓“两大任务”，着力加快项目建设。坚持把实施招商引资和项目争取“两大任务”作为加快项目建设，拉动投资发展的重大举措，全力推进，确保全社会固定资产投资增速不低于25%。全年计划实施各类重点项目473个，总投资753亿元，年内计划完成投资265亿元。其中，实施工业项目95个，农林水牧项目129个，城市建设项目125个，道路交通项目14个，商贸流通和旅游项目24个，社会事业及其他项目86个。加快推进211国道灵武至甜水堡及其连接线古窑子至青铜峡新材料基地高速公路建设，开工建设红寺堡至桃山高速公路。继续开展好文化宣传周及系列招商引资洽谈活动，积极承接东部产业转移，突出招商引资重点，主动外出到重点区域，围绕能源化工、食品加工、包装印刷等特色产业开展项目推介，不断扩大招商成果。筛选、包装、推介一批市场潜力大、竞争力强、经济效益好的大项目和好项目，以更大的力度、更硬的措施和更灵活的办法，面向国内外大企业集团招大商、引大资。力争全年招商落地资金突破150亿元，项目履约率、开工率均达到85%以上。科学编制“十二五”规划，认真储备和谋划一批“十二五”期间重点项目，力争全社会固定资产投资增幅年均增长25%以上。

(二)强化工业主导地位，着力发展新型工业。工业是我市经济发展的主力，也是受金融危机冲击最严重的领域。突出工业主导地位，坚定不移实施“兴工强市”战略是保持经济快速发展的关键所在。全年规模以上工业增加值增长16%以上，地方工业增加值增长20%以上。提升园区支撑能力。加快太阳山和盐同红三县(区)工业园区建设，把太阳山打造成新能源、镁合金和煤化工产业基地，年内投资2亿元，开工建设污水处理厂等12个基础设施项目，园区功能配套面积达到120平方公里。力争年内实现工业产值30亿元。提高青铜峡新材料基地、金积、嘉宝工业园区综合配套服务功能，完善盐池东顺、同心羊绒、红寺堡工业园区基础设施，规划建设利通区工业园区，努力形成竞相发展的新格局。壮大优势特色产业。做大做强煤炭、电力、化工等战略主导产业，改造提升电解铝、金属镁、硅铁、水泥、造纸等传统产业，着力培育光伏发电、风力发电、多晶硅等新兴产业。加快实施神华宁煤金凤矿井、庆华四股泉矿井等煤炭开采项目和青铝集团自备电厂、大唐新能源光伏发电、中节能光伏发电二期等能源电力项目，新增光伏、风力发电装机容量30万千瓦。力促庆华二期煤焦化、华能煤电一体化、国电吴忠热电公司热电联产、太阳镁业镁合金二期等项目开工建设，积极与宁东能源基地对接，做好下游产业链转移承接工作。加大中小企业扶持力度，促其发展壮大。努力壮大轻工业，实施佳美纸业生活用纸、科冕葡萄酒等项目，扩大轻工业在工业经济中的比重。提升骨干企业竞争力。实施产业优化升级、节能减排、技术创新和品牌创建等“四大工程”，加快西夏水泥、圣花米来扩建、双维绒业公司绒制品和宁龙塑胶管业板材生产技改等项目建设，努力实现优势骨干企业量的扩张和质的提升。力争新增销售收入过亿元的企业12家，过10亿元的企业3家，培育中国名牌产品1个，自治区名牌产品8个。加强对工业发展的协调服务。把做好对企业的服务作为推进工业发展的重要着力点，建立市直部门负责人深入企业绩效报告等制度，重点解决好企业发展中遇到的融资难、用地难、用工难、拆迁难等问题，帮助企业坚定发展信心，改善发展环境，实现更快发展。

(三)强化农业基础地位，大力发展现代农业。围绕建设滨河现代农业产业区、扬黄节水高效农业产业区、旱作避灾生态农业产业区的目标，积极推进农村土地流转，提高农业集约化程度。全市农业总产值达到65亿元，增长6%。推进“十大基地”上规模、上水平。实施“优质粮食、高产奶牛、高效温棚”等示范工程，建设孙家滩、叶升等现代农业示范园20个，发展优质粮食100万亩，种植有机水稻6万亩，冬麦20万亩，发展订单农业50万亩；新建奶牛规模养殖场、养殖大户110个，奶牛存栏达到17万头；新增滩羊30万只，饲养量530万只；新增肉牛5万头，出栏35万头，新增设施农业5万亩、高酸苹果5000亩、葡萄5万亩、人工甘草21万亩、红枣7万亩，发展适水产业2万亩，稻田养蟹1万亩。推进农业产业化经营。坚持“扶优、扶强、扶大”的原则，整合资源、资金、人才和技术，尽快形成带动能力强的产业龙头和企业集群，提高农业产业化经营水平。实施涝河桥牛羊肉市场深加工配套、中侨农牧肉牛育肥等项目；整合乳品资源，重点扶持夏进乳业、恒枫乳业扩规模、创品牌，培育两个产值分别过10亿元和5亿元的大型乳业集团。做强万胜、米来等一批骨干农产品加工企业，增强其龙头带动能力。完善农产品市场体系。加快农产品专业市场建设力度，组建农产品销售行业协会，发展壮大农村专业合作社，形成龙头企业+专业合作组织+基地(农户)的产业链条。加快无公害农产品、绿色农产品和有机食品示范基地建设及认证工作，设立无公害、绿色农产品和放心农产品专销区20个。争取国家星火计划、自治区重大科技项目，攻克优势特色产业重点领域关键技术难题，挖潜改造提升吴忠国家农业科技园区科技示范档次和水平。完善创新服务机制，加大农业科技人才培训力度，鼓励科技专业技术人员进入园区和企业创业。加强基础设施建设。继续坚持大规模高标准抓好农田水利基本建设，实施利通区、青铜峡市中央财政小型农田水利建设重点县项目，续建新建扁担沟扬水泵站更新改造等8项农田水利工程，实施同心、盐池、红寺堡节水补灌工程，发展节水补灌面积10万亩，实施杏树梁淤地坝工程等5项生态水利工程，治理水土流失面积200平方公里。加快农村道路建设，新建改建农村公路750公里，川区所有行政村通公交，山区92%的行政村通公交。

(四)突出特色优势，加快发展现代服务业。坚持把壮大第三产业，特别是发展具有吴忠特色和优势的现代服务业作为壮大地方财源、拉动消费增长的重要任务，完善基础配套，加大政策引导，力促有新的发展和突破。做强商贸流通业。围绕打造区域性商贸服务业中心目标，加快构建与多元化消费相吻合、与产业发展相配套、与城乡建设相适应的商贸服务体系。加大市场整合力度，加快“双百市场工程”和“三新工程”建设，开工建设东郊市场迁建二期工程。依托现有各类专业市场功能，打造牛羊肉集散、瓜果蔬菜集散、粮油加工购销集散、钢材建材集散、家具装饰材料集散和生活用品集散“六大物流基地”。完善农村流通服务体系，实行农产品流通企业与专业合作社直采对接，做好家电下乡、汽车摩托车下乡等管理工作，培育交易额亿元专业市场6家、5亿元专业市场3家，实现社会消费品零售总额51.2亿元。打造精品旅游线路。大力推进青铜峡黄河大峡谷旅游区建设，加快青铜古镇、黄河圣坛等重点项目开发建设步伐，完善金沙湾、108塔、牛首山寺庙群配套设施，争取进入5A级景区行列。整合提升市区、青铜峡黄河水上项目旅游圈，完善盐池革命烈士纪念园、同心红军西征纪念馆和红寺堡宁夏移民博物馆等景点功能，丰富红色旅游内容，构建青铜峡精品旅游、市区滨河风景旅游、盐同红色旅游圈，形成南接沙坡头、北连沙湖大旅游格局。力争全年接待游客145万人次，实现旅游总收入5.7亿元，完成“中国优秀旅游城市”创建目标。

(五)紧抓黄河金岸新机遇，再掀城乡建设新热潮。全面贯彻落实自治区建设沿黄城市带、打造“黄河金岸”的战略部署，深化“三城联创”成效，大力实施城乡基本建设，加快推进城乡一体化进程。加快滨河新区建设。瞄准建设滨河生态水韵城市目标，加快实施市区西移、青铜峡城市东扩战略，大规模开发建设黄河两岸30平方公里新区。重点抓好黄河公路大桥及桥头堡、黄河砌护工程、黄河楼、黄河生态星级酒店和“三广场一中心”等标志性工程建设。加快新区房地产项目开发，启动龙河唐都、圣花苑等38个房地产项目，大力配合实施211国道、灵甜线、古青线、京藏高速公路吴忠收费站迁移等道路建设项目，加快S101三期改造及市区灵州大道、利宁街南段等14条道路建设项目，构建快速交通路网。加快新区商贸、金融、医疗、电信等居民生活必须的服务网点布局，吸引人流、物流、资金流往新区聚集，为把滨河新区建成“黄河金岸”最大的亲水居住区打好基础。加快旧城改造步伐，加大环境整治和绿化、美化力度，重点解决交通、供热、供水等问题，完善城市功能。大力提升县城品位。利通区、青铜峡市围绕建成滨河首善之区和争创全国百强县目标，力争率先在城乡一体化上取得突破。红寺堡区围绕打造吴忠增长极、宁夏崛起区目标，加快新区规划及开发建设，大面积植树铺绿，加快生态建城步伐。盐池县围绕打造宁夏靓丽的东大门目标，加快实施县城东改西移、北控南扩，大力推进县城西区开发建设，做足生态文章、特色文章和资源文章。同心县按照打造中部干旱带上具有浓郁塞上风情的旱塬明珠目标，加快县城西移南扩，全力抓好县城新区3平方公里开发建设。太阳山按照建设水保生态工业城的目标，坚持产业开发和生态建设并举，提升湿地开发和保护利用水平。建设一批特色村镇。按照统筹城乡发展的总体目标，做好小城镇规划，完善基础设施，提高管理水平，强化城镇功能，美化城镇环境，提升综合承载力，打造金积、叶升等10个现代文明小城镇。加大村庄集中度建设，改善农民居住环境，新建“塞上农民新居”示范点14个，整治旧庄点70个。

(六)加大生态建设力度，努力推进环境友好型发展。生态建设功在当代、利在千秋。要把实现可持续发展作为最终追求，加快生态城市、生态农业、生态工业建设步伐，促进生态文明。生态城市建设再增亮点。依托现有黄河水系，挖掘湖泊湿地资源，实施黄河堤防景观绿化工程和高速公路两侧土地开发整理项目，青铜峡市和盐池县加快创建国家园林城市，红寺堡区、同心县加快创建自治区园林县城，全市建制镇要加快创建园林城镇。市区建设秦渠北岸、同心街等8条道路宽幅绿化带。完成城市绿化1万亩，新增城市绿地3000亩，新增水面6000亩，城市绿地率、绿化覆盖率分别达到36.5%和38.5%，园林式单位(小区)达到60%以上。完成黄河金岸绿色长城造林绿化1万亩，贯通黄河两侧辐射2公里的生态防护林体系。生态农业建设再上水平。实施农业综合开发、中低产田改造、土地整治等重点项目，加快三北四期、封山育林和防沙治沙重点建设，结合农田水利基本建设，完善农田防护林体系，加大农田林网、村庄绿化、绿色通道等防护林建设力度，全市全年完成造林绿化30万亩。建设高标准农田林网1万亩，村庄绿化1万亩，封山育林6.5万亩，治沙造林6.2万亩，新增林木育苗5000亩。以农村小康环保行动为抓手，强化水源地保护，治理面源污染，做好“一池三改”，实施村庄环境综合整治项目17个，规划农村饮用水源地24个，农村垃圾处理率达到70%，积极争创国家和自治区级环境优美乡镇和生态村。循环经济发展再求突破。实施昊胜造纸废水深度处理等20个节能减排项目，开展自治区级循环经济试点企业4家，市级6家。主要污染物二氧化硫和化学需氧量排放总量控制在7.17万吨和3.36万吨。城市环境空气质量二级以上天数较上年有所增加，黄河吴忠过境段段面水体水质稳定在三类，清水沟、南干沟水体达到五类水质标准。

(七)深入推进改革创新，为加快发展增添新动力。把改革作为前进的持续动力，把创新作为发展的不竭源泉，坚持不懈，大胆推进。向改革要活力。加快行政管理体制、经济领域、社会事业等各项改革，务求在一些重点领域和关键环节取得突破。进一步理顺市与市辖区、市与直管县的职责权限，健全政府职责体系，确保市辖区改革权责到位，“省管县”改革配合到位，政府机构改革运转到位，乡镇机构改革服务到位，充分调动积极性。继续深化行政审批制度改革，切实精简和规范行政审批事项，优化审批流程，提高行政效率。深化投融资体制改革，规范开源信用担保公司和城乡建设投资公司运行机制。推进公用事业单位改革，将垃圾处理、中水回用等公用事业推向市场。深化农村改革，规范和完善农村土地承包经营权流转，逐步扩大推广范围。实施保障和促进科学发展土地管理新机制、城乡建设用地增减挂钩、统筹城乡发展三项改革试点工作，严格执行土地利用总体规划和年度利用计划，认真落实集约节约用地制度，全面推进土地开发整理，确保占补平衡。积极探索城乡户籍制度改革，逐步打破城乡户籍屏障。向创新要动力。围绕新型工业、现代农业、现代服务业发展需求，鼓励和引导市场主体引进新技术、新工艺，提倡自主创新，提升产业核心竞争力。市本级财政年度安排500万贴息资金用于支持企业技术创新。积极落实与宁夏大学、宁夏医科大学等科研院校建立的产学研合作机制，力争取得实质性进展。建立科技信息、科技创新和科技培训体系，提高科技支撑能力。年内新培育5个企业技术中心、10个科技型企业。全市科技特派员队伍稳定在300人，发展一批专业技术人员进入企业创业。继续优化政务、司法、金融三大环境，重点解决服务效能低和建设项目落实地难、群众怕打官司和打官司难、信用缺失和融资难等问题。

（八）加强社会事业建设，着力保障和改善民生。民生连着民心，民心凝聚民力。解决好民生问题，让群众得到更多发展实惠是政府的根本责任。坚持优先发展教育。健全完善义务教育免费政策和经费保障机制，继续巩固提高“两基”成果。加快实施校舍安全工程，改善办学条件。红寺堡区、同心县实现教育强县(区)目标。启动利通区、青铜峡市普及高中阶段教育工作。大力发展职业教育，高度重视学前教育和特殊教育。深入开展名师名校名校长评选活动，不断提高教育教学水平。大力发展卫生事业。按照做大市级医院，做强县级医院，做实乡镇、社区和村卫生服务机构，做活民营医院的思路，加强市、县、乡、村四级医疗服务网络建设，完成吴忠市中医院、青铜峡人民医院和利通区胜利镇社区卫生服务中心等项目建设，打造15分钟医疗服务圈。强化食品、药品监管工作，确保食品、药品安全。加强计划生育工作。深入开展计划生育“星级乡镇”创建活动，实施“少生快富”工程，狠抓服务体系和信息化建设，加强流动人口计划生育管理服务，全市人口自然增长率控制在10.6‰，出生政策符合率达到88%以上。繁荣文化体育事业。制定出台加快特色文化产业发展意见，创新文化事业和文化产业体制机制。加强公共文化服务基础设施建设，加快文化信息资源共享工程和农家书屋建设。加强文化市场监管，进一步净化文化市场环境。全面实施《全民健身条例》，配套建设全民健身示范广场、乡镇全民活动中心，广泛开展全民健身活动，提高全民身体素质。高标准办好建城历史庆祝系列活动。健全社会保障体系。积极推进城镇居民、农民工、被征地农民、新型农村养老保险制度改革。启动利通区、青铜峡市新型农村养老保险自治区级试点工作。启动城镇职工基本医疗保险和生育保险市级统筹试点工作。积极探索全区范围内异地就医购药联网结算方法，努力实现医疗保险“一卡通”。抓好城乡低保、救灾救济、大病救助和社会临时救助工作，城乡特困户特重大疾病患者年医疗救助金额提高到5万元。探索养老服务新形式，促进居家养老社会化建设。继续大力改善农村特困群众居住条件，完成农村危房改造7100户，加快推进74个村“整村推进”步伐，搬迁移民2900户1.23万人，山区贫困人口减少15%。抓好创业就业工作。落实积极的创业就业政策，扎实开展创业型城市创建活动，落实小额担保贷款、税费减免、社保补贴等优惠政策。增加政府购买公益性岗位数量，重点帮助“零就业”家庭和就业困难人员，实现就业750人。加强职业技能培训和农村劳动力素质培训。全年完成职业技能培训1.65万人，城镇新增就业1.15万人，输出劳务25万人次，实现劳务收入10亿元。集中力量为民多办实事。加大城市廉租房、经济适用房建设力度，新建廉租房450套、经济适用房3万平方米，力争将城市低收入家庭全部列入廉租房保障。切实加强供水、供热、供气等基础设施建设，提升群众生活满意度。加快实施安全饮水工程，解决10万人的安全饮水问题。落实好市政府承诺为民办理的实事。加强民主法制建设。依法实行民主决策、民主管理、民主监督。建立和完善民意表达机制，畅通民主渠道。深入开展普法依法治理工作，研究制定“六五”普法规划。加强法律援助工作，满足困难群众法律需求。加强政府法制工作，提高政府法制透明度和公众参与度，做到规范、公正、文明执法。提升社会管理水平。严厉打击各类刑事犯罪，增强群众安全感。深入开展禁毒人民战争，遏制毒品犯罪势头。加强社会治安管理，推进社会管理创新，深入开展平安创建活动。切实加强安全生产监督管理，认真开展道路交通、消防、矿山等重点领域的专项整治，坚决遏制重特大事故的发生。构建应对突发性事件的危机管理机制。加强信访工作，强化信访工作责任，完善信访工作机制，依法规范信访秩序，认真对待群众诉求，着力解决信访突出问题。加强精神文明建设。强化思想道德建设，加强社会主义核心价值观体系建设，把人民群众的积极性、创造性凝聚到推进跨越式发展上来。大力加强未成年人思想道德建设，不断提高公民道德水平，形成良好的社会道德风尚。开展民族团结“示范社区”、“示范乡镇”创建活动。加强民兵、预备役部队及人民防空建设，完善国防动员体系，深入开展双拥共建工作。加强地震、档案、外事、统计、物价、广播电视、妇女儿童、残疾人事业、住房公积金等事业。

四、政府自身建设

各位代表！新的形势和任务，对政府工作提出了新的更高要求。各级政府及其工作人员必须在谋事、干事、干成事的能力上有新提升，在便民、帮民、富民的效率上有新突破，为确保完成全年各项目标任务，实现跨越式发展提供坚强保障。

努力建设学习型政府。读书学习是政府工作人员适应时代发展需要、提高综合素质、增强服务才干的重要途径。市人民政府将要求和引导各级政府工作人员尤其是领导干部，把学习当作一种修养、一种觉悟、一种境界，自觉带头爱读书、读好书、善读书，用理论武装头脑。坚持和发扬理论联系实际的学风，自觉运用理论指导实践。坚持从群众中来、到群众中去，充分尊重群众的原创精神，向群众学、向基层学、向老同志学。在各级政府当中努力形成学习的风气、调研的风气、探索的风气、实践的风气。通过开展各种形式的读书学习活动，有效解决理论不深、业务不精、水平不高、能力不强的问题，不断提高政府工作人员干事创业和应对复杂局面的能力，驾驭市场经济和服务科学发展的能力，努力打造学习型政府。

扎实推进依法行政。全面推进法治政府建设。规范公务员的行政行为，做到合法行政、合理行政、程序正当、高效便民、诚实守信、权责统一。深入贯彻行政许可法，继续推行行政审批制度改革，减少行政许可和审批事项，特别要减少投资审批、项目核准，落实企业的投资主体地位。促进公平竞争，消除地区封锁，打破行业垄断，组织调动各种社会资源，促进经济增长。加强对工程项目、财政资金使用、产权交易、土地出让、政府采购等重点领域和关键环节的监督，规范行政行为。不断完善拆迁安置政策，不再新欠农民征地补偿费。继续强化制度建设和落实，把不作为、乱作为和严重损害群众利益等行为作为问责重点，严肃追究给国家利益、公共利益和公民合法权益造成严重损害的行为。

坚持科学民主决策。继续把依法决策、科学决策、民主决策贯穿始终，做到程序依法规范、过程民主公开、结果科学公正。对政府重大决策的形成和执行，加强调查研究，做到察民情、听民意、聚民智，尊重客观规律，提高决策的预见性、科学性和有效性。进一步落实重大事项专家咨询、社会公示、听证、质询制度，让群众知道政府在想什么、做什么，保障人民群众的知情权、参与权、表达权、监督权。自觉接受市人大及其常委会的法律监督和工作监督，市政协的民主监督、新闻舆论监督和社会公众监督，完善和加强政府内部监督。认真办理人大代表建议和政协委员提案。建立公众评议机制，加强群众监督和舆论监督。

扎实推进作风转变。要求和引导各级政府工作人员牢固树立为基层服务、为企业服务、为群众服务的意识，坚持工作前置、重心下移，加强调查研究，做到问政于民、问需于民、问计于民，多深入困难问题多、群众意见多、工作瓶颈多的地方，千方百计为企业排忧，满腔热情为群众解难，真心实意为基层办事。进一步强化抓落实的责任，做到各项工作责任到人，有部署、有检查，一盯到底。大力倡导勇于直面矛盾、善于破解难题、敢抓敢管敢负责任的作风，坚决反对花拳绣腿、坐而论道、脱离群众，坚决消除虎头蛇尾、推诿扯皮、敷衍塞责，勇于担当，不畏艰险，愈挫愈勇，争创一流。

不断强化制度建设。继续深化人事制度改革。认真落实学习培训、岗位轮换、考核等制度，提高公务员履职、服务能力，激活用人机制，最大限度地发挥人力资源效率。完善事业单位编制管理与聘用人员控制数管理相结合的新机制，盘活事业编制资源。全面推行事业单位绩效工资改革。扎实落实党风廉政建设责任制，深入开展“小金库”专项治理，强化对国有资产的规范管理。实行公务费“零增长”管理，严格规范政府采购。今年政府投资力度大、新上项目多，要确保监管到位。要强化监察、审计等专门监督，高度重视群众监督和舆论监督，做到行政权力运行到哪里，监督就落实到哪里，财政资金运行到哪里，审计就跟进到哪里，严防发生腐败现象。

各位代表！时代赋予重任，奋斗铸就辉煌。让我们高举中国特色社会主义伟大旗帜，紧密团结在以胡锦涛同志为总书记的党中央周围，在市委的坚强领导下，全面贯彻落实科学发展观，坚定信心，昂扬奋进，攻坚克难，扎实工作，为实现吴忠跨越式发展而努力奋斗！
